# Linking inflammation, metabolic dysfunction, and neurodegeneration: a comprehensive review of TLR2 pathways in type 2 diabetes

**DOI:** 10.3389/fcdhc.2026.1791782

**Published:** 2026-03-23

**Authors:** Juan Antonio Arreguín-Cano, Sandra Aidé Santana-Delgado, Carlos Esteban Villegas-Mercado, Grissel Guadalupe Orozco-Molina, Adolfo González-Acosta, Mercedes Bermúdez

**Affiliations:** Faculty of Dentistry, Autonomous University of Chihuahua, Chihuahua, Mexico

**Keywords:** chronic low-grade inflammation, immunometabolism, insulin resistance, neurodegeneration, neuroinflammation, toll-like receptor 2 (TLR2), type 2 diabetes mellitus (T2DM)

## Abstract

Type 2 diabetes mellitus (T2DM) is a chronic metabolic disorder increasingly recognized as a systemic inflammatory condition with significant neurological effects. Growing evidence shows that chronic low-grade inflammation (CLGI), insulin resistance, and metabolic imbalance contribute to cognitive decline and the development of neurodegenerative diseases like Alzheimer’s and Parkinson’s. Toll-like receptor 2 (TLR2), a critical pattern-recognition receptor of the innate immune system, has emerged as an essential molecular link between metabolic dysfunction and neuroinflammation and neuronal damage. This review summarizes current experimental, clinical, and translational evidence on the role of TLR2 in T2DM-related inflammation, mitochondrial dysfunction, lipid imbalance, insulin resistance, and blood–brain barrier (BBB) issues. We explore how ongoing TLR2 activation by internal danger signals and metabolic stressors maintains systemic inflammation and fuels neuroimmune responses via microglial activation and cytokine release, thereby accelerating neurodegenerative processes. Additionally, we discuss new therapeutic strategies targeting TLR2 signaling, including drugs, dietary supplements, and the repurposing of antidiabetic medications with neuroprotective effects. By combining immunometabolic and neurodegenerative pathways, this review highlights TLR2 as a promising target for preventing or reducing diabetes-related cognitive decline neurodegeneration.

## 1 Introduction

T2DM is a major global health issue. In 2021, about 529 million had diabetes, with 96% T2DM, accounting for 95% of related disability-adjusted life years (1). The increasing impact of T2DM on disability and death rates is significant (2). Elevated body mass index is recognized as the leading risk factor for T2DM, responsible for 52% of the global disease burden (1, 3). By 2050, the global number of people affected by diabetes is expected to exceed 1.3 billion, with particularly high rates projected for regions including the Middle East, North Africa, and Latin America (1, 4). The geographic differences in diabetes rates and the rising burden in low- and middle-income countries highlight the urgent need for better prevention strategies, early detection tools, and effective public health measures (5).

The connection between metabolic dysfunction and inflammation is essential to the development and progression of various neurodegenerative diseases, including Alzheimer’s disease (AD) and Parkinson’s disease (PD) (6). Obesity, often resulting from diets high in fat and genetic factors, contributes to common metabolic conditions like T2DM and metabolic dysfunction-associated steatotic liver disease (MASLD) (6, 7). Moreover, increasing evidence shows that these metabolic disruptions play a major role in triggering neuroinflammatory and neurodegenerative diseases (6, 8). Cohort studies in AD and PD patients have shown a link between diabetes and a higher risk of developing both diseases, with a 1.21-fold increase in the risk of idiopathic PD among patients with T2DM (9). Excessive nutrient intake and resulting fat gain lead to metabolic problems, showing as disrupted energy balance and reduced insulin sensitivity in the liver, along with a persistent systemic inflammation indicated by high cytokine levels (6, 10). Hyperglycemia has been shown to damage pericytes in the blood-brain barrier (BBB), which can cause “leakage” that makes the brain vulnerable to toxins and peripheral inflammatory mediators (9). Lipids such as ceramide and palmitate, along with lipid transporters like lipocalin-2 (LCN-2) and apolipoprotein E (ApoE), are key components contributing to neuroinflammation (11). Elevated levels of palmitate, a fatty acid, have been detected in the cerebrospinal fluid of obese individuals with mild cognitive impairments. Palmitate can activate brain immune cells, such as astrocytes and microglia, leading to TNF release and fostering an inflammatory brain environment (8, 12). Dysfunction of LCN-2, a protein that helps regulate brain inflammation, has been linked to weakened BBB integrity and activation of inflammatory signaling pathways like NF-κB, leading to increased production of pro-inflammatory cytokines (12, 13). Animal studies have shown that inefficiencies in this interaction may lead to obesity, glucose intolerance, and neurodegenerative diseases, and may even trigger a dysfunctional and self-perpetuating “metabolic triangle” marked by insulin resistance, mitochondrial dysfunction, and brain inflammation (12, 14). Mitochondrial dysfunction, characterized by a significant decrease in complex I activity, is a key pathological factor in PD, making dopaminergic neurons in the substantia nigra particularly vulnerable due to their high ATP demands (9).

TLR2, a member of the toll-like receptor family, plays a critical role in initiating and regulating immune and inflammatory responses. It is widely expressed on various peripheral innate immune cells, including neutrophils, monocytes, macrophages, dendritic cells, and natural killer cells (15). Within the brain, TLR2 is also found in neurons, endothelial cells, and glial cells, including microglia, astrocytes, and oligodendrocytes (16). This strategic distribution highlights its potential role as a link between the immune system, metabolism, and neurological functions (16, 17). The field of immunometabolism reveals complex interactions between metabolic mechanisms and inflammatory responses. Immune cells in the periphery and CNS undergo metabolic reprogramming during immune responses to meet energy needs and synthesize defense molecules (18, 19). TLRs, by recognizing pathogen-associated molecular patterns (PAMPs), trigger innate immune responses by regulating cellular metabolism. For example, lipopolysaccharide (LPS) binding to TLRs has been linked to metabolic reprogramming from mitochondrial oxidative phosphorylation (OXPHOS) to aerobic glycolysis in dendritic cells (human *ex vivo/in vitro*) (18, 20). Accelerated glycolysis is crucial for quick ATP production and nucleotide synthesis in many leukocytes, whereas anti-inflammatory responses tend to shift toward OXPHOS (19). This review aims to compile current evidence supporting TLR2’s role in shared inflammatory signaling pathways and to assess its potential as a therapeutic target for preventing or mitigating diabetes-related neurodegenerative processes.

## 2 Methods

This study was a structured narrative review aimed at providing a mechanistic and conceptual synthesis of the relationship among type 2 diabetes mellitus (T2DM), Toll-like receptor 2 (TLR2), metabolic inflammation, and neurodegeneration. A comprehensive literature search was conducted across PubMed/MEDLINE, Scopus, Web of Science, and SciELO. No strict lower date limit was imposed in order to incorporate foundational mechanistic studies; however, most included references span approximately 2010 to 2025, reflecting advances in immunometabolism and neuroinflammatory research. Search strategies combined controlled vocabulary and free-text terms, including “Type 2 Diabetes Mellitus”, “T2DM”, “Toll-like receptor 2”, “TLR2”, “chronic low-grade inflammation”, “insulin resistance”, “mitochondrial dysfunction”, “blood–brain barrier”, and “neurodegeneration”, adapted to each database.

Eligible studies included original experimental research (*in vitro* and animal models), human observational studies, and translational investigations examining TLR2-related inflammatory or metabolic mechanisms relevant to neurological outcomes in T2DM. Articles not directly addressing TLR2 signaling pathways or lacking methodological clarity were excluded from the core synthesis. Screening was conducted through iterative evaluation of titles, abstracts, and full texts based on conceptual relevance. Data extraction focused on convergent inflammatory and metabolic signaling pathways, including MyD88/NF-κB activation, oxidative stress, mitochondrial dysfunction, insulin signaling impairment, BBB disruption, and microglial activation, as well as associated clinical and imaging findings (**Table 1**).

**Table 1:** PRISMA-like overview of literature identification and selection (Structured narrative review).

Stage	Description
Type of review	Structured narrative review (conceptual and mechanistic synthesis integrating preclinical and clinical evidence; not a systematic review or meta-analysis).
Databases searched	PubMed/MEDLINE, Scopus, Web of Science, and Scielo.
Timeframe of literature considered	No strict lower date limit was imposed to allow inclusion of foundational mechanistic studies. The review integrates seminal and contemporary literature, with the majority of cited references spanning approximately 2010 to 2025, reflecting advances in immunometabolism, TLR signaling, and neurodegeneration research.
Search focus/key concepts	Type 2 diabetes mellitus; TLR2; CLGI; immunometabolism; insulin resistance; mitochondrial dysfunction; oxidative stress; BBB disruption; neuroinflammation; microglial activation; AD; Parkinson’s disease; cognitive decline.
Study types considered	Human observational studies (cross-sectional, cohort, epidemiological); experimental animal models; *in vitro* mechanistic studies; translational and interventional studies when available.
Inclusion approach	Studies were included based on relevance to TLR2 signaling in metabolic dysfunction, inflammatory pathways, cerebrovascular alterations, or neurodegenerative processes in the context of T2DM. Both mechanistic and clinical data were considered when conceptually linked.
Exclusion approach	Articles not directly addressing TLR2-related mechanisms, metabolic inflammation, or neurological outcomes in diabetes were not considered central to the narrative synthesis. Editorials, commentaries, and studies lacking methodological clarity were excluded.
Screening strategy	Iterative, concept-driven screening was conducted to support mechanistic integration rather than exhaustive quantitative selection. Titles and abstracts were screened for relevance, followed by full-text review when necessary.
Data extraction	Qualitative synthesis of molecular pathways (MyD88/NF-κB, MAPK, inflammasome activation), metabolic outcomes (insulin resistance, lipid dysregulation, mitochondrial dysfunction), neurovascular mechanisms (BBB integrity), and clinical correlates (cognitive decline, imaging biomarkers).
Assessment of bias/quality	A formal quantitative risk-of-bias assessment was not performed. However, methodological limitations, heterogeneity across models, and translational constraints are explicitly discussed in the manuscript, with careful distinction between preclinical mechanistic evidence and human observational findings.

Although a formal quantitative risk-of-bias assessment was not performed, methodological rigor, study design, and translational relevance were carefully considered in the interpretation. A clear distinction was maintained between mechanistic evidence derived from preclinical models and associative findings from human studies.

## 3 TLR2 and inflammatory pathways in type 2 diabetes

T2DM is characterized by chronic LGI, which is significantly affected by the activation of innate immune receptors, such as TLR2 (21). This chronic inflammatory state, which is intrinsically linked to insulin resistance, significantly affects immune cell function, leading to dysregulation of both pro-inflammatory and anti-inflammatory responses (22). Such immune dysfunctions contribute to increased susceptibility to infections and various complications seen in diabetic individuals (23). Furthermore, the persistent LGI characteristic of T2DM is now recognized as a key factor in its development and progression, shifting the understanding of this condition from solely a metabolic disorder to one with critical inflammatory components (24).

### 3.1 Role of TLR2 in recognizing DAMPs and PAMPs

TLR2 recognizing DAMPs triggers downstream signaling cascades involving myeloid differentiation primary response 88 and NF-κB leading to the transcription of pro-inflammatory cytokines such as interleukin-1β (IL-1β) (25–27). This cytokine plays a crucial role in modulating insulin secretion and contributing to β-cell apoptosis, exacerbating the pathology of T2DM (25). Furthermore, elevated levels of TLR2 expression have been observed in individuals with obesity and T2DM, correlating with increased serum levels of IL-18, a marker of inflammatory response (28). Beyond this, TLR2 activation by saturated fatty acids, such as palmitate, has been shown to induce insulin resistance in myotubes by rapidly associating myeloid differentiation factor 88 with the receptor, thereby activating stress-activated kinases such as p38, JNK, and protein kinase C (29).

**Figure 1:**
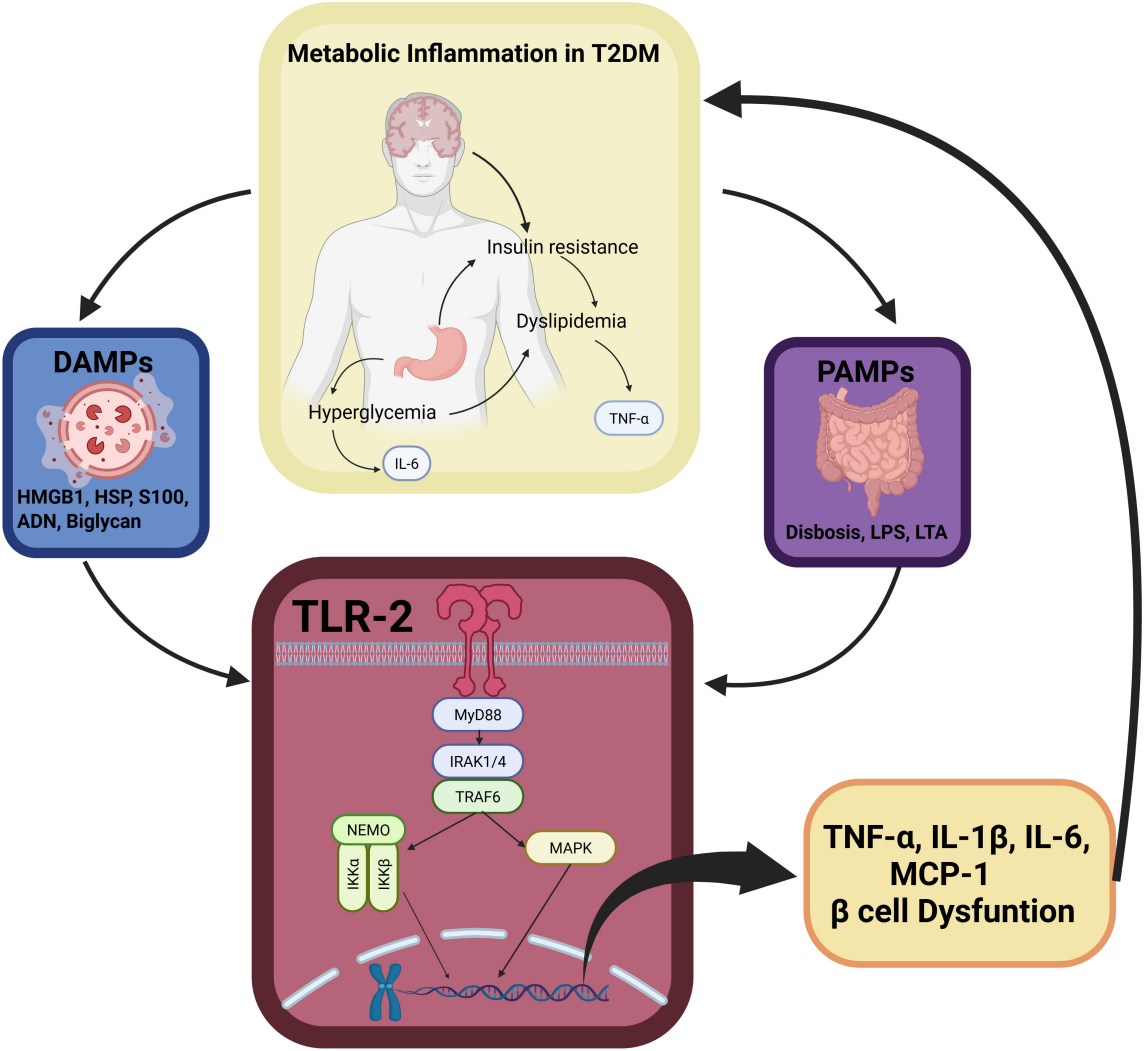
Schematic representation of Toll-like receptor 2 (TLR2) as central immunometabolic hub linking endogenous DAMPs, damage-associated molecular patterns; PAMPs, pathogen-associated molecular patterns. Metabolic stressors (hyperglycemia, dyslipidemia) activate TLR2 signaling through MyD88-NF-κB and MAPK pathways, driving transcription of pro-inflammatory cytokines (TNF-α, IL-6, IL-1β) and MCP-1, monocyte chemoattractant protein-1. This establishes a feedforward loop amplifying insulin resistance and pancreatic β-cell dysfunction. Curved arrows depict bidirectional crosstalk sustaining chronic low-grade inflammation characteristic of type 2 diabetes mellitus pathogenesis.

TLR2, which recognizes PAMPs derived from microbial components, also triggers robust immune responses, further implicating the gut microbiota in the genesis of metabolic inflammation observed in obesity and T2DM (30). Indeed, dysbiosis of the gut microbiota, a common feature among obese and diabetic individuals, significantly contributes to systemic inflammation by raising the circulating levels of microbial products that activate TLR2 (31). This activation subsequently triggers complex intracellular signaling pathways, such as the NF-κB and activator protein-1 cascades, leading to the transcriptional upregulation of pro-inflammatory cytokines and acute-phase inflammatory mediators (32). These pathways not only enhance systemic inflammation but also directly disrupt insulin signaling, aiding in the development and ongoing progression of insulin resistance (33, 34). The interaction between TLR2 and lipopolysaccharides (LPS) from Gram-positive and Gram-negative bacteria, along with long-chain fatty acids, notably stimulates the activation of mitogen-activated protein kinases (MAPK) and Janus N kinase, further exacerbating insulin resistance (35) (**Figure 1**).

### 3.2 TLR2 and the activation of NF-κB and downstream pro-inflammatory cytokines (TNF-α, IL-1β, IL-6)

This complex interaction ultimately results in increased expression of key inflammatory markers, such as TNF-α, which further spreads systemic inflammation and interferes with insulin signaling pathways (32). This ongoing inflammatory environment then hampers insulin signaling and cellular glucose absorption, ultimately worsening insulin resistance (36). The persistent activation of these pathways contributes to the overall inflammatory burden in T2DM, creating a vicious cycle that further compromises metabolic homeostasis (37, 38). Furthermore, this ongoing inflammatory environment triggers the activation of inflammasomes, such as the NOD-like receptor protein 3 inflammasome, which converts pro-IL-1β and pro-IL-18 into their active forms, fueling a cycle of inflammation that directly leads to pancreatic β-cell dysfunction and insulin resistance (39, 40). Given this established role, TLR2 signaling is also closely linked to the upregulation of IL-6, a pleiotropic cytokine that further worsens systemic inflammation and insulin resistance in T2DM patients (41).

## 4 TLR2-mediated crosstalk between metabolic dysfunction and neurodegeneration

TLR2, a key immunometabolic regulator in T2DM, links innate immune activation to alterations in cellular energy balance, lipid metabolism, and insulin signaling. In metabolically active tissues, persistent TLR2 stimulation by endogenous danger signals maintains low-grade inflammation that gradually impairs mitochondrial function, lipid metabolism, and glucose use, leading to insulin resistance and metabolic deterioration. The following subsections outline the main molecular mechanisms by which TLR2 contributes to metabolic dysfunction in T2DM.

### 4.1 TLR2 and metabolic dysfunction

#### 4.1.1 Mitochondrial dysfunction and oxidative stress

Mitochondrial dysfunction and oxidative stress are key effects of TLR2 activation in metabolic tissues, connecting innate immune responses to cellular energy failure in T2DM (42). Engagement of TLR2 by endogenous ligands, such as saturated fatty acids, AGEs, and damage-associated molecular patterns (DAMPs), activates downstream pathways including MyD88–NF-κB and MAPKs (43). This signaling increases the production of pro-inflammatory cytokines (TNF-α, IL-6, IL-1β) and inducible nitric oxide synthase, which disrupts the mitochondrial electron transport chain activity (44). As a result, electron leakage from complexes I and III increases the production of reactive oxygen species (ROS), while ATP synthesis efficiency declines (45). Excess ROS leads to oxidative modification of mitochondrial proteins, peroxidation of membrane lipids, and damage to mtDNA, all of which further weaken mitochondrial integrity and promote fragmentation. In β-cells, which already have low antioxidant capacity, this oxidative stress speeds up apoptosis and hampers insulin secretion (46). In peripheral tissues, mitochondrial dysfunction reduces fatty acid oxidation and glucose use, worsening insulin resistance. Importantly, studies show that pharmacological or genetic inhibition of TLR2 decreases ROS production and maintains mitochondrial function in models of diet-induced obesity and diabetes, highlighting its potential as a therapeutic target to break the cycle of inflammation, oxidative stress, and metabolic decline (47–49).

#### 4.1.2 Lipid metabolism and free fatty acid signaling

Dysregulated lipid metabolism and increased circulating free fatty acids (FFAs) in obesity and T2DM directly activate TLR2, generating a pro-inflammatory metabolic environment (50). FFAs act as endogenous danger signals that enhance TLR2 expression and downstream signaling in adipocytes, hepatocytes, and macrophages (51). This leads to increased release of TNF-α and IL-6, attracts pro-inflammatory macrophages into adipose tissue, and promotes lipolysis. As a result, ectopic fat builds up in skeletal muscle and liver, causing steatosis and lipotoxic injury. At the cellular level, TLR2 signaling worsens endoplasmic reticulum stress and apoptosis when combined with excess lipids, further impairing metabolic health dysfunction (52). Conversely, studies in Tlr2 knockout mice show decreased hepatic lipid accumulation and enhanced systemic lipid management under a high-fat diet conditions (45). Thus, FFAs and TLR2 signaling act synergistically, linking dyslipidemia with chronic inflammation and insulin resistance in T2DM (22, 53, 54).

#### 4.1.3 Impact on glucose uptake and insulin sensitivity

TLR2 activation hampers glucose regulation by disrupting insulin receptor signaling in muscle, fat, and liver. Activation of TLR2 leads to downstream kinases, such as JNK and IKKβ, which phosphorylate insulin receptor substrate (IRS) proteins on inhibitory serine residues (55, 56). This modification inhibits PI3K–Akt activation, decreases GLUT4 translocation to the plasma membrane, and lowers insulin-stimulated glucose uptake in skeletal muscle and adipocytes (57). In the liver, TLR2 signaling promotes gluconeogenesis while reducing insulin’s ability to suppress glucose production, thereby worsening hyperglycemia. Notably, animal studies show that mice genetically deficient in Tlr2 (Tlr2^-^/^-^) are protected against insulin resistance caused by a high-fat diet, with better glucose tolerance and insulin sensitivity (42). Furthermore, blocking Tlr2 with drugs restores insulin response in cell and animal studies (58). These findings identify TLR2 as a crucial mediator connecting innate immune activation, metabolic inflammation, and disrupted insulin signaling in T2DM.

### 4.2 TLR2 in neurodegeneration associated with type 2 diabetes

#### 4.2.1 Shared inflammatory pathways between T2DM and AD

T2DM and AD share similar inflammatory pathways, with TLR2 serving as a key mediator. In T2DM, persistent hyperglycemia, insulin resistance, and dyslipidemia activate low-grade systemic inflammation via NF-κB and MAPK signaling, leading to ongoing cytokine release, including IL-6 and TNF-α (59, 60). Similarly, in AD, amyloid-β aggregates bind to TLR2 on microglia, triggering MyD88-dependent pro-inflammatory cascades that worsen oxidative stress and synaptic damage dysfunction (61). These shared mechanisms contribute to impaired neuronal insulin signaling, mitochondrial dysfunction, and cognitive decline, leading some authors to propose AD as type 3 diabetes (59). Evidence from both human and animal models supports the idea that metabolic inflammation in T2DM prepares the brain for neurodegeneration, while amyloid pathology increases inflammatory damage. Therefore, TLR2-mediated signaling acts as a molecular link connecting systemic metabolic issues to neuroinflammatory processes that drive AD progression.

#### 4.2.2 BBB disruption and microglial activation

**Figure 2:**
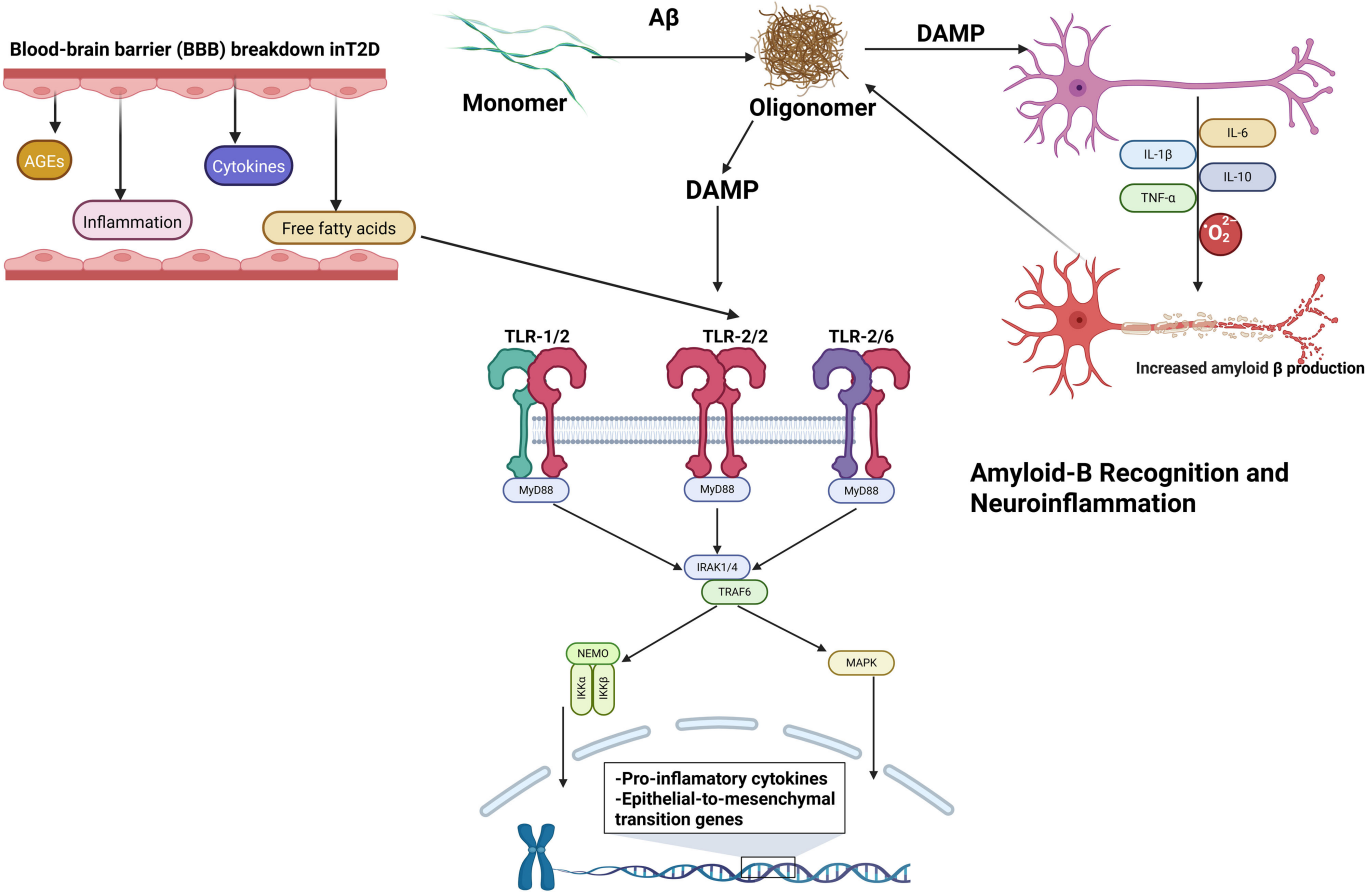
TLR2 mediates neuroinflammatory progression in diabetes-associated neurodegeneration by linking peripheral metabolic inflammation (FFAs/cytokines via BBB breakdown) to central amyloid-β (Aβ) recognition on microglia, activating MyD88-NF-κB/MAPK signaling that transcriptionally amplifies pro-inflammatory cytokines and establishes self-perpetuating Aβ oligomerization and synaptic dysfunction.

The BBB is especially susceptible to metabolic stress in T2DM, where chronic hyperglycemia, advanced glycation end products (AGEs), and inflammatory mediators raise endothelial permeability (62). TLR2 signaling contributes to this dysfunction by promoting vascular inflammation, disrupting tight junctions, and enabling infiltration of peripheral immune cells into the brain parenchyma (63). Once compromised, the BBB exposes neurons to circulating cytokines and FFAs, further increasing oxidative stress. In the central nervous system (CNS), TLR2 on microglia is activated by amyloid-β and other damage-related molecules, promoting their shift toward a pro-inflammatory state phenotype (64). Ongoing TLR2 activation thus connects systemic metabolic inflammation with local neuroimmune responses, worsening cognitive decline T2DM (65). Targeting TLR2 could therefore maintain BBB integrity and reduce harmful microglial activation in neurodegenerative conditions (**Figure 2**).

#### 4.2.3 Animal and human evidence linking TLR2 overexpression to cognitive decline

Numerous studies highlight the significant increase of TLR2 expression and activity in diabetic patients, linking it to higher levels of pro-inflammatory cytokines such as TNF-α, IL-6, and IFN-γ, which contribute to the development of insulin resistance and diabetic complications (44). Experimental evidence shows that Tlr2 deficiency in animal models reduces inflammation and improves glucose regulation, indicating its crucial role in T2DM development (45). Targeting TLR2 signaling pathways could be a promising approach for managing type 2 diabetes by reducing inflammation and improving metabolic outcomes. Hyperinsulinemia and hyperglycemia, characteristic features of T2DM, can also increase TLR2 expression, further amplifying inflammation and insulin resistance (29, 46, 47). This bidirectional relationship creates a feedback loop that promotes T2DM progression (29). Evidence from animal and human studies links Tlr2 overexpression to cognitive decline associated with T2DM and AD. In mice, Tlr2deletion reduces amyloid-β–induced neurotoxicity, preserves synaptic plasticity, and improves cognition (66). Conversely, TLR2 overexpression or chronic stimulation accelerates neurodegeneration, with increased microglial activation and neuronal loss (67). In humans, elevated TLR2 levels in blood monocytes and brain tissue correlate with systemic inflammation and cognitive impairment in T2DM and AD. Studies also suggest that higher TLR2-related inflammatory markers are associated with accelerated hippocampal atrophy and worsening cognitive trajectories (65, 68–71). These findings underscore TLR2 as both a mechanistic contributor and potential biomarker of diabetes-related neurodegeneration, making it a candidate target for therapies aimed at slowing or preventing cognitive decline.

#### 4.2.4 Potential overlap with Parkinson’s disease and other neurodegenerative disorders

TLR2 signaling has been linked not only to AD but also to PD and other neurodegenerative disorders (72). Misfolded α-synuclein, the hallmark of PD pathology, acts as a TLR2 ligand, activating microglia and promoting the release of pro-inflammatory cytokines that lead to dopaminergic neuronal loss in the substantia nigra (73). Experimental evidence demonstrates that inhibiting TLR2 reduces α-synuclein–induced neurotoxicity, supporting its role in disease development. Similar mechanisms are proposed in Huntington’s disease and amyotrophic lateral sclerosis, where misfolded proteins might activate innate immune pathways via TLR2 (74). The co-occurrence of T2DM with these disorders suggests that metabolic inflammation exacerbates TLR2-driven neuroinflammatory cascades, accelerating disease progression (75). Thus, TLR2 acts as a standard immunometabolic hub that links systemic metabolic dysregulation with central neurodegeneration. Therapeutically, strategies targeting TLR2 could have wide-reaching use across various neurodegenerative diseases beyond AD, providing a unified approach to reduce inflammation-driven neuronal loss.

## 5 Clinical evidence linking TLR2, diabetes, and neurological outcomes

### 5.1 Human studies on TLR2 expression in peripheral monocytes and CNS-related biomarkers.

In human studies, TLR2 expression on monocytes is significantly elevated in patients with Type 1 Diabetes Mellitus who have microvascular complications (26, 76). Similar increases in TLR2 expression have been observed in monocytes and neutrophils from patients with T2DM (26, 77, 78). Elevated TLR2 levels in clinical samples from patients with diabetes mellitus-associated atherosclerosis have been correlated with increased pyroptosis and higher concentrations of inflammatory cytokines such as IL-1β and IL-18 (26).

While direct human studies linking peripheral monocyte TLR2 expression to CNS biomarkers are ongoing, evidence highlights TLR2’s role in neuroinflammation and cognitive issues in diabetes. TLR2 contributes to diabetic vascular disease and cognitive impairment, possibly via cerebrovascular dysfunction causing reduced blood flow and cognitive deficits (79). Activation of TLR2 can trigger neuroinflammation, impairing spatial learning and fear conditioning (79). Moreover, in a Mayo Clinic Study of Aging cohort, diabetes status was found to affect levels of CSF inflammatory molecules, displaying age-related correlations with AD biomarkers such as total tau, phosphorylated tau-181, neurofilament light chain, and YKL40 (80).

### 5.2 Inflammatory markers and imaging evidence of neurodegeneration

Diabetic patients often show abnormal brain structure and function, influenced by oxidative stress, inflammation, and mitochondrial dysfunction that impair synaptic transmission and plasticity, leading to neuronal and cognitive deficits. Elevated highly activated microglia are observed in the hippocampus of patients with diabetes (81).

Inflammation plays a key role in diabetic retinopathy, as evidenced by inflammatory changes in retinas and vitreous humor of diabetic animals and patients. Anti-inflammatory agents benefit in preventing vascular and neuronal issues over time. Inflammatory cytokines like IL-1β, IL-6, IL-8, TNF-α, and MCP-1 are elevated in serum and eye samples from diabetic patients with retinopathy (82).

**Figure 3:**
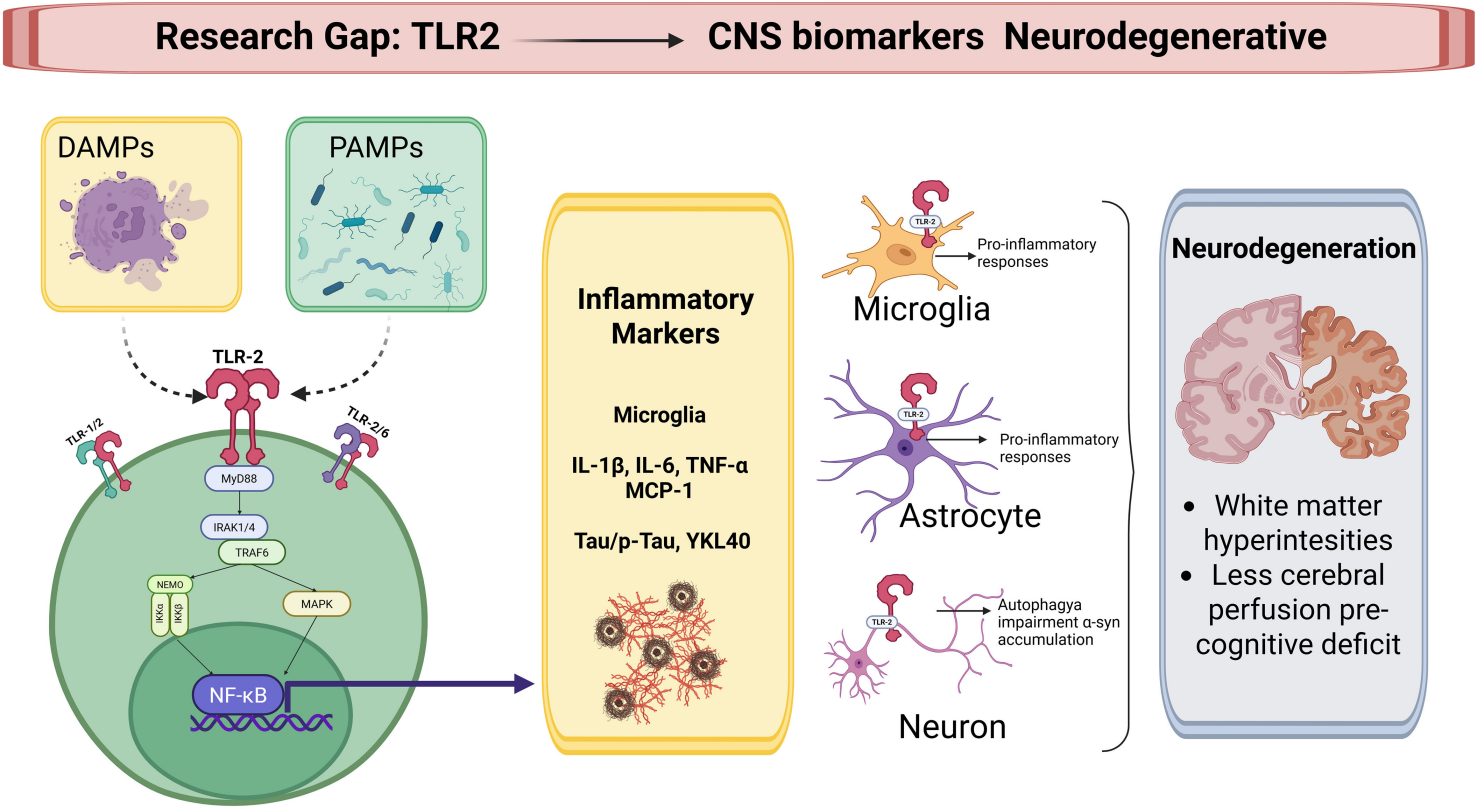
TLR2-driven neuroinflammatory research gap: peripheral DAMPs/PAMPs activate monocyte TLR2 expression → central microglial/astrocyte activation releases IL-6/TNF-α → CSF biomarkers (Tau, phosphorylated Tau, neurofilament light chain, YKL-40) correlate with neuroimaging findings of white matter hyperintensities, hippocampal atrophy, and reduced cerebral perfusion preceding cognitive deficits in T2DM.

Regarding imaging evidence, changes in vascular function and cerebral perfusion are known to occur before cognitive deficits in diabetes (79). Studies have shown that localized atrophy in the hippocampus, a brain region essential for memory, may be mainly responsible for the memory deficits observed in diabetic populations, with hippocampal volume decreasing as the duration of diabetes increases (81). Patients with T2DM may also show brain atrophy and white matter hyperintensities (83). Specific white matter volumes in areas like the posterior cingulate, precuneus, insula, and rostral middle frontal gyrus have been proposed as independent imaging biomarkers to detect cognitive impairment in T2DM patients (84) (**Figure 3**).

Biomarkers often examined in studies of diabetes mellitus-related cognitive decline include C-reactive protein, tau protein, brain-derived neurotrophic factor, AGEs, glycosylated hemoglobin, and adipokines (85). Elevated peripheral BDNF levels, especially when associated with reduced insulin secretion, may predict memory problems in patients with long-term T2DM (86). While these studies highlight the role of serum inflammatory markers and imaging in neurodegeneration in T2DM, a detailed correlation between specific markers and imaging findings in humans is still being explored.

## 6 Therapeutic implications

### 6.1 Preclinical studies on TLR2 antagonists and inhibitors.

Preclinical research shows targeting TLR2 could reduce diabetes-related neurological issues. Tlr2^-^/^-^ mice in a Type 1 Diabetes model were protected from reduced brain perfusion and cognitive problems, such as long-term fear learning impairments in the hippocampus and prefrontal cortex (79). This genetic deletion also prevented the development of endothelium-dependent vascular dysfunction associated with diabetes (79). Furthermore, Tlr2^-^/^-^ mice showed protection against insulin resistance and beta cell dysfunction induced by a high-fat diet (87).

Pharmacological strategies targeting TLR2 have also demonstrated promise. In rodent models, direct inhibition of Tlr2(e.g., via intravenous injection of anti- Tlr2 antibody) or indirect modulation of TLR2 and TLR4 signaling (e.g., using the dual cholinesterase inhibitor DL0410) has been shown to reduce neuroinflammation and enhance cognitive functions (88). In an experimental cell model of diabetic mellitus-associated atherosclerosis, Tlr2 inhibition reduced elevated TLR2 levels and prevented activation of the MyD88/NF-κB signaling pathway, which was confirmed to improve inflammation and atherosclerosis in diabetic mice in an *in vivo* study (26). These findings suggest that Tlr2 could be a promising therapeutic target for diabetic mellitus-associated atherosclerosis (26). Additionally, a TLR2 antagonist has been shown to ameliorate T2DM-associated neuropathic pain by repolarizing pro-inflammatory macrophages (89).

While TLR2 is a primary focus, related TLR pathways also present treatments. *In vitro*, blocking TLR4 (which forms heterodimers with TLR2) using agents like L48H37 (a curcumin analog) or C20 (a natural flavonoid) can restore oligodendrocyte maturation and myelination by reducing inflammation and nitroxidative stress (88). Pharmacological inhibition of Tlr4 has also been shown to protect against both acute and chronic fat-induced insulin resistance in rats (90). A selective Tlr4 inhibitor, CLI-095, effectively blocked human and murine TLR4 signaling and prevented autoimmune diabetes in NOD mice (91). Acute TLR4 inhibition with eritoran has even been shown to improve insulin resistance in humans (92).

### 6.2 Nutraceuticals and natural compounds modulating TLR2 activity

Nutraceuticals and natural compounds are studied for their potential to modulate TLR2 activity and offer neuroprotection in diabetes. For example, the curcumin analog L48H37 and flavonoid C20 inhibit TLR4, reducing inflammatory cytokines and nitroxidative stress, restoring oligodendrocyte function *in vitro* (88). Polyphenols are also being studied as new treatment options for diabetes-related neuropathy, emphasizing the need for safe and effective alternatives to traditional therapies (93).

Natural herbal medicines and their bioactive ingredients have anti-inflammatory and antioxidant effects, reducing hyperglycemia symptoms and slowing diabetic complications. Some natural compounds help by inhibiting the NLRP3 inflammasome (94). Specific neuroprotective phytochemicals such as Aromatic-turmerone, Apocynin, and Matrine are considered promising candidates for developing novel treatments against neurodegenerative diseases (95). The use of bioactive compounds that can decrease intracellular protein aggregation in models of neurodegenerative disorders may also help alleviate symptoms of T2DM (96). Furthermore, redox-modulating and immunomodulating nutraceuticals are appealing options for preventing and treating inflammation, including in diabetes (97).

### 6.3 Potential repurposing of anti-inflammatory and anti-diabetic drugs targeting TLR2 pathways

Reusing anti-inflammatory and anti-diabetic drugs to target TLR2 pathways could help manage neurological outcomes in diabetes. Since neuroinflammation and diabetes involve pathways like NLRP3 inflammasome and IL-1β, drugs with anti-inflammatory effects might be repurposed for quicker clinical use (98). Several anti-diabetic medications are being studied for their neuroprotective effects (**Table 2**).

**Table 2:** Anti-diabetic medications with neuroprotective potential.

Medication/Drug class	Neuroprotective mechanism	Key findings	Type of study	Primary references
Sulfonylureas (e.g., gliquidone; glibenclamide)	Modulation of innate immune signaling and NLRP3 inflammasome activity in glial cells	Gliquidone attenuated LPS-induced inflammatory activation in BV2 microglia and primary astrocytes via NLRP3-related pathways	*In vitro* + Animal model	(99)
Empagliflozin (SGLT2 inhibitor)	Preservation of neurovascular unit integrity; attenuation of ultrastructural remodeling	Reduced neurovascular remodeling and glial alterations in db/db diabetic mice	Animal model	(100)
Insulin (central signaling)	CNS insulin signaling modulates neuroinflammation and neuronal survival pathways	Mechanistic review supports anti-inflammatory and neuroprotective effects; basis for therapeutic repurposing	Translational review (mixed: *in vitro* + animal + human data)	(101)
Metformin	Anti-inflammatory, antioxidant, and mitochondrial regulatory effects	RCT in amnestic MCI showed cognitive and metabolic imaging outcomes; meta-analysis associated metformin with reduced dementia risk in T2DM	Human data	(102, 103)
Thiazolidinediones (PPAR-γ agonists)	PPAR-γ–mediated anti-inflammatory and metabolic modulation	Phase III rosiglitazone trial showed no overall benefit but subgroup signals; pioglitazone prevention trial negative for delaying MCI onset	Human data (Phase III clinical trials)	(104, 105)
SGLT2 inhibitors	Reduction of high-glucose–induced inflammatory toxicity in microglia	Canagliflozin attenuated inflammatory signaling and cytotoxicity in BV-2 microglia under hyperglycemic conditions	*In vitro*	(106)
α-glucosidase inhibitors (acarbose)	Metabolic modulation influencing neuroinflammation and aging-related pathways	Chronic acarbose improved behavioral and biochemical markers in SAMP8 mice; ameliorated metabolic-cognitive alterations in 3xTg-AD model	Animal models	(107, 108)
GLP-1 receptor agonists (liraglutide)	Enhancement of neurotrophic signaling and reduction of neuroinflammation	ELAD trial evaluated liraglutide in mild-to-moderate AD; translational data support synaptic and inflammatory modulation	Human data	(109, 110)
DPP-4 inhibitors (sitagliptin; linagliptin)	Incretin-mediated neurotrophic signaling; reduction of microglial activation	Sitagliptin associated with cognitive outcomes in elderly T2DM patients; linagliptin reduced microglial activation in diabetic mice	Human data + Animal model	(111, 112)

While these approaches are promising, it is important to note that despite the potential, trials specifically aiming to repurpose anti-diabetic drugs for late-onset dementia have not yet consistently demonstrated success in improving cognitive decline (113). The central role of TLRs in innate immunity and the development of diabetes and its complications makes them important drug targets. Strategies aimed at reducing TLR activity could potentially eliminate inflammation in diabetes and lower related complications (114–116). While these strategies are scientifically compelling, most evidence for TLR2 modulation comes from preclinical studies in cell cultures or animals. Deletion or blockade of TLR2 has improved insulin sensitivity and reduced inflammatory markers in mouse models of metabolic disease (58), yet translation to human clinical benefit remains uncertain. More broadly, anti-inflammatory interventions targeting innate immune pathways have shown variable or limited cognitive benefit in human neurodegenerative disease trials, underscoring the translational gap between experimental neuroprotection and clinical outcomes (117).

Importantly, systemic inhibition of TLR2 raises safety concerns. Disruption of TLR signaling pathways has been linked to increased susceptibility to infection and changes in immune balance in experimental models (118). Furthermore, innate immune receptors such as TLR2 and TLR4 share inflammatory pathways and interact with downstream inflammasome activation, especially NLRP3 (117, 119), suggesting that broad inhibition of a single receptor may cause compensatory or unintended neuroimmune effects.

These considerations suggest that therapeutic strategies should focus on selective, context-dependent modulation rather than complete receptor blockade. Targeting specific downstream signaling adaptors, heterodimer configurations, or cell-type–restricted expression within the neurovascular unit may improve translational accuracy while preserving essential innate immune functions.

Although TLR2 is highlighted in this review as a key receptor connecting metabolic dysfunction and neurodegeneration, it operates within a broader innate immunometabolic network that includes other pattern recognition receptors such as TLR4, the receptor for AGEs, and the NLRP3 inflammasome. Recent evidence shows that TLR4 signaling is triggered by metabolic stressors, including high glucose levels and metabolic DAMPs, and it promotes NF-κB–mediated inflammation and cytokine production in both metabolic tissues and the CNS (120). Similarly, the AGE–RAGE axis is upregulated in diabetes and interacts with common downstream pathways, including TLR4 and MyD88/NF-κB signaling, increasing chronic inflammation and oxidative stress responses relevant to diabetic complications and neurodegeneration (121). The NLRP3 inflammasome functions as an integration point downstream of TLR and RAGE signaling, leading to caspase-1 activation and the maturation of IL-1β and IL-18, which contribute to neuroinflammatory injury in models of neurodegenerative disease (122). Additionally, comprehensive reviews on innate immunity in Alzheimer’s and Parkinson’s disease highlight the complex interaction among these pathways, where innate receptors coordinate both protective and harmful immune responses in the brain (123).

Within this context, TLR2 warrants specific emphasis for its ability to recognize a broad spectrum of endogenous ligands, such as lipid derivatives and DAMPs that increase in metabolic disorders. Unlike TLR4 and RAGE, which respond mainly to endotoxin and glycation products respectively, TLR2’s distinct pattern recognition capabilities and its expression across various cell types — from peripheral immune cells to microglia and endothelial cells — place it at a key junction between systemic immunity and neuroimmune signaling. Together, these receptors and pathways form a coordinated immunometabolic network rather than acting as isolated factors. A balanced view that places TLR2 within this larger framework respects its unique contributions while recognizing the overlapping and cooperative roles of TLR4, RAGE, and NLRP3 in chronic inflammation and neurodegenerative diseases.

## 7 Knowledge gaps and future directions

Despite increasing evidence linking TLR2 signaling to inflammation, metabolic dysfunction, and neurodegeneration in T2DM, key knowledge gaps still exist that hinder therapeutic progress. Most importantly, there is a lack of large-scale, long-term human studies capable of clarifying causality, progression, and the timeline of cognitive decline (124, 125). Current research is limited by small clinical sample sizes, which weaken statistical power and generalizability (28, 113, 114), and by predominant cross-sectional designs, which preclude causal inferences (126–128). Translational hurdles also loom large, including the lack of integrated models that connect systemic metabolic disruptions to CNS outcomes (129), unresolved mechanisms of cerebral glucose dysregulation and insulin resistance (81), inadequate exploration of cell-specific TLR2 expression in the neurovascular unit (88), and unaddressed complexities like the obesity paradox in stroke and cognition (129). Bridging these gaps requires thorough, multi-modal research to develop TLR2-targeted interventions.

A deeper understanding of how TLR2 activation in the cerebrovasculature contributes to cognitive impairment in T2D is essential, especially regarding its impact on endothelium-dependent vascular function and cerebral blood flow (130). While some studies suggest that reduced regional cerebral perfusion in T2DM patients is linked to cognitive decline, overall cerebral blood flow might not differ substantially, highlighting the importance of more detailed investigations into specific microvascular changes rather than general assessments (131). Additionally, the exact signaling mechanisms underlying altered cerebrovascular communication with the CNS in obesity and chronic diseases such as T2DM remain largely unexplored (132). Future research should also examine how diabetes-related changes affect specific steps of the neurovascular coupling cascade and clarify the direct impact of pathological alterations on NVC measurements (129, 133).

One of the main issues is the lack of large-scale longitudinal studies in humans, which are essential for understanding how cognitive impairment progresses in T2DM (125, 134, 135). Similarly, existing studies often have relatively small clinical sample sizes, which can limit statistical power and the generalizability of findings (26, 136, 137). This limitation reduces the reliability of outcomes and highlights the need for larger, more diverse groups to improve the generalizability of research findings and strengthen the evidence for clinical recommendations. Cross-sectional designs, common in much of the current literature, further impede the ability to establish causal links between diabetes and cognitive decline, emphasizing the need for longitudinal data from larger samples (126–128). Future research should also include multi-wave designs in cohort studies to better evaluate the timing and relationships among different risk factors and their effect on cognitive decline (124). Another important area needing further study is how metabolic diseases influence the risks and outcomes of ischemic stroke and cognitive impairment, especially considering the “obesity paradox” where higher stroke risk in obese individuals contrasts with better outcomes in experimental models (129). This paradox highlights the complexity of metabolic disease mechanisms and calls for more research into different physiological responses at various disease stages and among diverse populations.

There is also a need for translational models that integrate metabolic and neurological outcomes. These models would allow for a more comprehensive understanding of how systemic metabolic dysfunction interacts with CNS health, moving beyond isolated organ-focused approaches. Additionally, the roles of abnormal glucose transport and intracellular glucose metabolism in the brain require thorough investigation to clarify their contributions to cognitive dysfunction in diabetes (81). Specifically, researchers should examine how impaired cerebral insulin signaling, a key pathogenic mechanism in regulating glucose metabolism, leads to reduced ATP production, increased oxidative stress, and inflammation, thereby promoting neuronal damage and cognitive impairment in diabetic patients and animal models (81). Future studies should also establish stronger correlations between functional network deficits and clinical outcomes by incorporating cognitive behavioral assessments and other parameters absent from prior research (138). Furthermore, investigating the fundamental mechanisms of diabetes-related brain damage, such as mitochondrial dysfunction, endoplasmic reticulum stress, and impaired autophagy, is crucial for discovering new therapeutic targets.

Future directions point to the potential of personalized medicine approaches targeting TLR2 signaling, such as the development of selective TLR2 agonists or antagonists tailored to individual patient profiles, especially given that TLR-mediated neuroinflammation plays a critical role in cognitive dysfunction (88). Further research into the precise cell-specific expression patterns of TLR2 within the neurovascular unit and its downstream signaling cascades will be vital for such targeted interventions (88). Moreover, longitudinal studies are essential to track TLR2-mediated neuroinflammatory progression and its correlation with cognitive decline, thereby informing early intervention strategies and therapeutic monitoring (139). Ultimately, this deeper understanding could enable the development of novel pharmacological tools to modulate TLR2 pathways, mitigating neuroinflammation and preserving cognitive function in individuals with T2DM (26, 140). However, complete TLR2 removal may not be wholly beneficial, as Tlr2^-^/^-^ mice display cognitive phenotypes resembling schizophrenia, including hyperlocomotion and attention deficits (141). This highlights the need for a delicate balance in Tlr2 modulation, as full ablation risks unintended neurodevelopmental or neuropsychiatric consequences rather than purely neuroprotective effects. Thus, future studies should explore strategies that selectively target downstream pathways or cell-type-specific TLR2 activation to maximize therapeutic benefits without disrupting essential physiological functions (142). A promising alternative involves ligands that selectively bind to TLR2 heterodimers (such as TLR2/1 or TLR2/6) to precisely regulate inflammatory responses relevant to diabetic neurodegeneration, considering the context-dependent effects of TLR activation (88). Such refined modulation could leverage TLR2’s beneficial roles in pathogen recognition and tissue repair while curbing its pro-inflammatory contributions to chronic neurodegeneration in T2DM (130, 143). Additionally, investigating the interaction between TLR2 and other innate immune receptors, such as the NLRP3 inflammasome, is essential, given their combined role in worsening diabetic inflammation and neurodegeneration (26). Finally, clarifying how hyperglycemia and hyperlipidemia, hallmarks of T2DM, directly affect TLR2 expression and signaling in glial cells will be key to targeted interventions against cognitive decline (144), particularly since TLR2 activation contributes to diabetic microvascular complications and is upregulated in patients with type 1 diabetes (26, 79). Future research should focus on developing specific small-molecule TLR2 inhibitors for preclinical testing in models of diabetes-related neurodegeneration, as well as on exploring optimal tissue-specific delivery methods to improve therapeutic accuracy (26).

## 8 Conclusion

TLR2 links metabolic dysfunction, chronic inflammation, and neurodegeneration in T2DM. Prolonged TLR2 activation causes insulin resistance, mitochondrial issues, BBB breakdown, microglial activation, and neuroinflammation. These processes explain increased vulnerability to cognitive decline and neurodegenerative diseases like AD and PD in T2DM. Targeting TLR2 could reduce metabolic and neurodegenerative damage without impairing immune functions, making it a promising therapy to disrupt the cycle between metabolic and neuroinflammatory issues in diabetes-related neurodegeneration.

## Glossary

AD: Alzheimer’s disease
AGEs: Advanced glycation end products
BBB: Blood–brain barrier
CNS: Central nervous system
DAMPs: Damage-associated molecular patterns
FFAs: Free fatty acids
IL: Interleukin
IRS: Insulin receptor substrate
CLGI: Chronic low-grade inflammation
MAPK: Mitogen-activated protein kinase
MASLD: Metabolic dysfunction-associated steatotic liver disease
NF-κB: Nuclear factor kappa B
NLRP3: NOD-like receptor family pyrin domain-containing 3
PAMPs: Pathogen-associated molecular patterns
ROS: Reactive oxygen species
T2DM: Type 2 diabetes mellitus
TLR2: Toll-like receptor 2
